# Holistic Review, Mitigating Bias, and Other Strategies in Residency Recruitment for Diversity, Equity, and Inclusion: An Evidence-based Guide to Best Practices from the Council of Residency Directors in Emergency Medicine

**DOI:** 10.5811/westjem.2022.3.54419

**Published:** 2022-05-10

**Authors:** Moises Gallegos, Adaira Landry, Al’ai Alvarez, Dayle Davenport, Martina T. Caldwell, Melissa Parsons, Michael Gottlieb, Sreeja Natesan

**Affiliations:** * Stanford University School of Medicine, Department of Emergency Medicine, Palo Alto, California; † Harvard Medical School, Department of Emergency Medicine, Boston, Massachusetts; ‡ Rush University Medical Center, Department of Emergency Medicine, Chicago, Illinois; § Henry Ford Health System, Department of Emergency Medicine, Detroit, Michigan; ¶ University of Florida College of Medicine – Jacksonville, Department of Emergency Medicine, Jacksonville, Florida; || Duke University School of Medicine, Department of Emergency Medicine, Durham, North Carolina

## Abstract

Advancement of diversity, equity, and inclusion (DEI) in emergency medicine can only occur with intentional recruitment of residency applicants underrepresented in medicine (UIM). Shared experiences from undergraduate and graduate medical education highlight considerations and practices that can contribute to improved diversity in the resident pool, such as holistic review and mitigating bias in the recruitment process. This review, written by members of the Council of Residency Directors in Emergency Medicine (CORD) Best Practices Subcommittee, offers best practice recommendations for the recruitment of UIM applicants. Recommendations address pre-interview readiness, interview approach, and post-interview strategies that residency leadership may use to implement holistic review and mitigate bias for recruitment of a diverse class.

## BACKGROUND

Efforts to advance diversity, equity, and inclusion (DEI) in medicine are dependent on deliberate attention toward residency recruitment.^1^,^2^ The benefits of diversity in medicine are well known, including cultural sensitivity and competence, expanded delivery of healthcare in low-resource settings, and improved intellectual discussion within training cohorts.^3^ It is important to develop pipeline pathways for racial/ethnic UIM groups^a^ to increase the number of residency candidates.^4^ A study of the 20 largest Accreditation Council for Graduate Medical Education (ACGME) resident specialties observed that no residency program represented Black or Latino populations at comparable rates to the United States population.^5^ For emergency medicine (EM), it was predicted to take 54 years to achieve a similar representative proportion of the Latino population.^5^ That study emphasizes the continued need to support diversity, equity, and inclusion through improved parity in medical clinician representation. While this article focuses on recruitment of UIM applicants, there are other populations that do not fall under the strict definition of UIM that are at risk of underrepresentation or exclusion, such as students from rural, LGBTQ+, or religious communities, and special consideration for these applicants should also be taken.^6^–^8^

Fundamental to UIM recruitment is recognition and mitigation of bias. While bias exists at all stages of recruitment, it is most notable in high-impact metrics such as the United States Medical Licensing Examination (USMLE) Step examinations, Standardized Letters of Evaluation (SLOE), the Medical Student Performance evaluations (MSPE), and induction into the Alpha Omega Alpha (AOA) Honor Medical Society.^9^–^11^ Holistic review emphasizes balanced consideration of these metrics with additional components such as personal statement, extracurricular activities, and lived experience.^12^,^13^ The impact of bias in the UIM recruitment process is consequential: applicants may be disregarded during pre-interview screening or ranked lower post-interview,^9^ further hindering the mission to increase diversity in medicine.^2^

Unfortunately, there is no standardized process to increase holistic review and minimize bias in resident selection. Using current literature, we outline best practice recommendations for implementing holistic review and mitigating bias in residency recruitment to promote DEI.

## CRITICAL APPRAISAL

This is the ninth article in a series of evidence-based best practice reviews from the Council of Residency Directors in Emergency Medicine (CORD) Best Practices Subcommittee.^14^–^20^ With the guidance of a medical librarian, we used MEDLINE via PubMed to search for articles published from inception to February 4, 2021, using keywords and medical subheadings focused on diversity, equity, and inclusion (Appendix). We searched the bibliographies of relevant articles for any additional studies. The search yielded 2080 articles, of which 115 were deemed to be relevant for inclusion in this review. Articles were independently screened by two authors who searched for those that address holistic review and bias in recruitment and interviewing. We included articles if either author recommended the relevance of the study. When supporting data was not available, recommendations were made based on the authors’ combined experience and consensus opinion. According to the Oxford Center for Evidence-Based Medicine criteria, we provide the level and grade of evidence for each best practice statement (Table 1).^21^ This manuscript was reviewed by the CORD Best Practices Subcommittee and posted to the CORD website for peer review and feedback from the CORD medical education community.

## PRE-INTERVIEW PREPAREDNESS STRATEGIES

### Defining DEI Goals for Recruitment Season

Increasing DEI efforts and improving the recruitment of UIM residency applicants requires purposeful planning at programmatic, departmental, and institutional levels.^2^,^6^,^22^–^25^ Advanced preparation ahead of the recruitment season can facilitate holistic review and contribute to decreasing bias in the selection process. Residency leadership should first define what diversity means for the program, including measurable outcomes and consequences of not achieving these results.^10^,^24^,^26^ A statement of purpose can aid tracking and accountability of progress toward set goals.^26^–^28^ Acknowledgment of DEI in mission statements demonstrates residency program investment in diversity as a core value.^2^,^6^,^24^ There should be a clear call for increased representation of UIM residents, including a definition of the role the institution will take.^27^,^29^ With early and clear communication of a commitment to, and goals for, diversity recruitment, residency programs can position themselves for success throughout the interview and selection process.

### Assess Program Readiness

Commitment to increasing the number of UIM residents is defined by the internal discussions and actions that address the value of diversity, not simply match results.^12^ Recruiting diverse residents should be included as part of a program’s strategic plan.^24^,^31^ Support from the department chair and organizational leadership is key to the success of diversity initiatives.^2^,^22^,^24^,^30^,^32^,^33^ Programs should undergo an internal review process of current culture toward and readiness to enact targeted recruitment efforts for DEI.^6^,^7^ To achieve meaningful success for DEI in recruitment and departmental attitudes, programs need to embrace and foster an environment of change.^31^,^34^ The Association of American Medical Colleges (AAMC) presents a four-step process for assessing institutional culture and climate (Table 2).^35^

Programs can also complete diversity engagement surveys to assess an institution’s level of engagement and inclusion, and perceptions within the group.^6^–^8^ Programs should work to increase awareness, interest, and engagement in DEI efforts through department-wide educational sessions.^2^,^33^ Programs should highlight how they will foster the career and academic interests of UIM trainees.^36^,^37^ Support should be proactive, such as assigning resident mentors early, asking UIM trainees about individual needs, and providing early in-service exam preparation for all matriculating residents with marginal USMLE and other assessment scores.^2^ Programs should avoid blindly targeting UIM trainees with services such as test prep, however, as this can reinforce bias and stereotypes.

### Formation of Diversity Committees

Programs should create diversity committees with an understanding of program goals and objectives for the recruitment of UIM applicants. Valuing UIM status during interview screening and selection and greater UIM faculty representation is a program characteristic associated with higher resident diversity.^38^–^40^ Committee members should be included in all planning phases of recruitment and should include UIM and non-UIM faculty, residents, and staff.^11^,^25^,^50^,^51^

The formation of a diversity recruitment committee can be impactful.^23^,^25^ In just one year, the Denver Health Emergency Medicine residency program doubled the number of UIM applicants interviewed, relying on a diversity committee to inform recruitment practices.^50^ Similarly, the Highland Emergency Medicine residency program in Oakland, CA, experienced a doubling of diversity representation in their residency group after implementation of recruitment initiatives spearheaded by a diversity committee.^52^ A diversity committee can have immediate and measurable impacts on UIM recruitment. It should be reiterated, however, that success in recruiting UIM candidates is most predicated upon the creation of a welcoming, supportive, and inclusive culture at the program, not just match statistics.

Representation of UIM faculty is integral to recruiting UIM resident applicants.^20^,^38^–^40^ Recruitment and retention of UIM faculty are discussed in a separate review as part of the CORD Best Practices series.^20^ Mindful attention should be made to not assign UIM faculty with work that is unaligned with their personal interests, underrecognized by promotions committee, and uncompensated despite the time investment. It is important to recognize the potential for UIM individuals to experience a “minority tax,” or disproportionate burden of work.^1^

### Accessing UIM Applicants

It is difficult to recruit diverse candidates, however, if they do not exist within the applicant pool. Recruitment can take on a variety of forms depending on the target populations and the desired messaging.^44^,^53^ Dedicated outreach to UIM students can lead to increased interest in a given program.^6^,^29^,^54^–^56^ Reaching UIM applicants requires more than just simple communication as programs need to demonstrate a commitment to diversity and service.^29^,^57^,^60^ Programs should display their commitment, efforts, and successes with DEI efforts on their websites,^2^,^6^,^25^ and should provide contact information for a point person, faculty or staff, to address questions about DEI within the program.

Recruitment can be enhanced through early enrichment and pathway programming.^57^–^59^ In addition to medical school interest groups, there may be a benefit to connecting with pre-medical organizations at the university level,^23^ and creating enrichment programs as early as the elementary and high school levels.^2^,^29^ The UIM applicants may not have personal or professional networks to initially steer them toward medical school and subsequently assist with residency applications.^60^ Early outreach can occur by way of faculty presence at dedicated conferences sponsored by UIM student organizations, and faculty volunteering as mentors through sponsored programs.^2^,^6^,^25^,^29^,^51^

An underused tool in UIM recruitment is a formal collaboration with minority medical student organizations,^61^ It should be clear, however, that a lack of attendance or participation with these groups should not affect the applicant’s consideration or rank-list position. The Student National Medical Association proposes a five-phase recruitment strategy using minority medical student organizations to increase the number of UIM students entering medical school. Increased matriculation of UIM medical students will directly contribute to the applicant pool for residency and the strategies suggested can be adapted to residency recruitment.^61^

**Best Practice Recommendations**

1. Define clear and prioritized goals for diversity-related residency recruitment. (Level 5, Grade D)
2. Assess program readiness to implement diversity-related recruitment and support UIM trainees that match. (Level 4, Grade C)
3. Mitigate bias through inclusion of bias training and predetermined scoring rubrics for screening, interviews, and ranking. (Level 3, Grade B)
4. Create DEI committees to inform and steer diversity-related recruitment. (Level 3, Grade B)
5. Ensure representation of UIM faculty in the screening, interview, and selection process but avoiding tasking UIM faculty with too much during the recruitment cycle. (Level 5, Grade D) (Level 4, Grade C)
6. Begin recruitment of UIM applicants early through directed and expanded efforts such as enrichment, outreach, and pathway programming. (Level 5, Grade D)
7. Collaborate with minority student groups in early mentorship and advisory programs for UIM applicants. (Level 5, Grade D)

## INVITATION AND INTERVIEW STRATEGIES

At every step of the process, programs should approach recruitment with a lens to promote diversity, ensure inclusion, support equity, and uncover and address biased and racist practices.^62^ Programs should go beyond simply recognizing bias, aiming to actively mitigate it, aligning with the ACGME Common Program Requirements to improve diversity.^63^ Individuals involved in recruitment, interviewing, and ranking should complete implicit bias training,^10^,^30^,^33^,^40^–^43^ and programs should conduct sensitivity discussions and self-reflection to promote learning about biases.^44^ Interviewers should undergo training and preparation as a group to decrease variability and bias in applicant evaluations.^48^

### Approach to Holistic Review

A standardized holistic review process that aligns with each institution’s mission, vision, and values will shift the focus away from a traditionally metrics-driven selection process to a more inclusive process. Holistic review focuses on the importance of the applicant and their stories, rather than achieving certain demographic numbers.^2^,^12^,^28^,^62^,^64^,^65^ As there is no universal approach to holistic review, it is important to recognize that the process is subject to bias as program leadership determines its implementation.^13^,^24^

**Figure 1:**
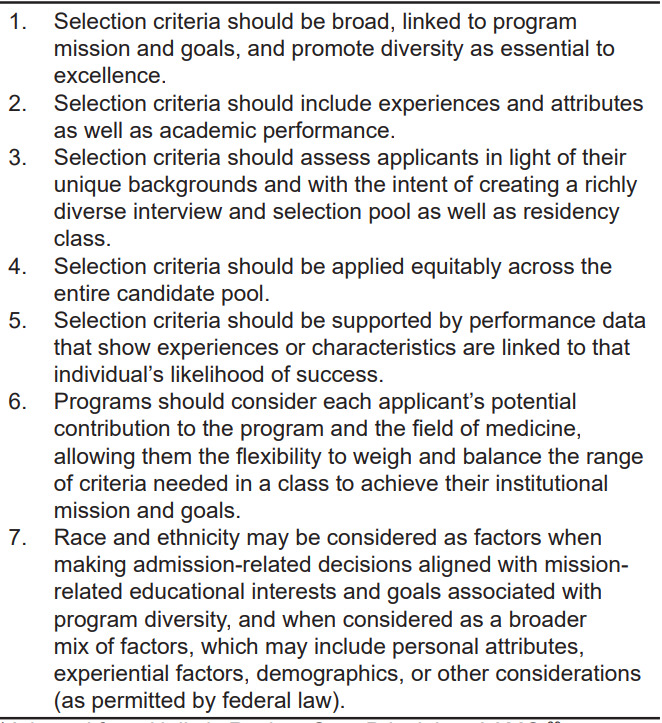
Core principles for holistic review.* *Adapted from Holistic Review-Core Principles, AAMC.^69^

Holistic review has more readily made its way into undergraduate medical education.^66^ Residency recruitment continues to rely heavily on performance and assessment metrics.^67^,^68^ Principles of holistic review in medical school admissions can be extrapolated to inform residency recruitment. The AAMC offers a holistic review primer for program directors to identify experiences, attributes, competencies, and metrics grounded in a program’s mission.^69^,^70^ The AAMC Advancing Holistic Principles Advisory Committee promotes core principles for holistic review (Figure 1).^69^

**Figure 2:**
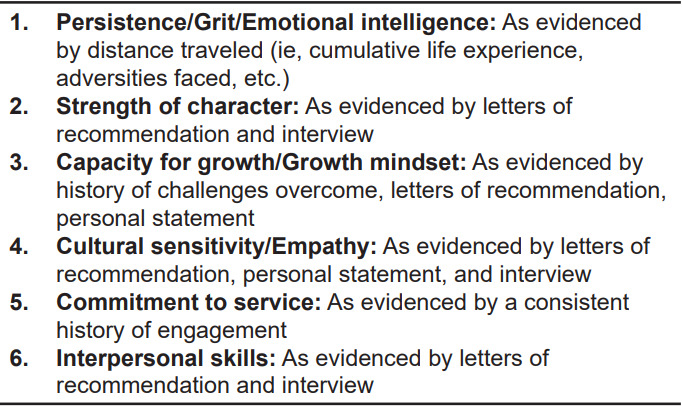
Qualities and characteristics to consider during holistic review.* *Adapted from DeBenedectis (2019) and Witzburg (2013).^12^,^73^

Holistic review addresses the need to balance personal attributes with performance and aptitude.^6^,^7^,^12^,^71^ It shifts the practice of preferentially valuing academic achievement-based metrics to considering the entire application.^2^,^6^,^7^,^10^,^64^,^71^ In this approach, numerical benchmarks, such as test scores and class rank, do not prematurely eliminate or accelerate applicants prior to the evaluation of the entire application.^25^ There have been different models of holistic review suggested in the medical education literature.^1^,^2^,^43^,^48^,^55^,^62^ Review committees should begin with a self-audit of current practices and make appropriate changes that best fit the program’s goals.^6^ Notably, the search for applicants who “align well” with a program, a concept known as “fit,” introduces bias that must be acknowledged and addressed.^43^ Figure 2 summarizes qualities and characteristics proposed for holistic review in place of traditional metrics. Over reliance on metrics such as exam scores and AOA status will impact recruitment of candidates who are underrepresented in medicine or systematically disadvantaged.^6^,^11^,^12^,^26^,^72^

The impact of holistic review on in-service and medical board examination pass rates is not yet well documented. Nehemiah et al demonstrated no significant change for surgical in-training exam scores after the implementation of holistic review and an accompanying increase in UIM diversity.^65^ Aibana et al involved stakeholders and committee members in deciding a new Step 1 threshold unlikely to affect board passing rates.^78^ Below we explore the value and harm of core components of the application and strategies to optimize a holistic review.

### Applicant Selection for Residency Interview

No single, uniformly accepted evaluation system exists for offering residency interviews, thereby allowing for subjectivity, bias, and inconsistency when selecting candidates.^7^,^11^,^26^,^74^ Scoring rubrics for all phases of recruitment, from interview selection to ranking, should be decided ahead of time.^47^,^75^–^78^ Rubrics should reflect the level of importance that experiences, attributes, competencies, or metrics represent for a program, and can help de-emphasize metrics that can bias selection against the UIM applicant.^77^,^79^ The AAMC provides a guided activity for Applicant Criteria Identification and Prioritization as part of its holistic review capacity building resources.^80^ Table 3 highlights examples of scoring rubrics that incorporate concepts of holistic review.

#### Clinical Grades and Letters of Recommendation

For EM applicants, their clinical evaluation hinges on the sub-internship SLOE. This summative form provides a rating and ranking of the student as well as descriptive commentary of their performance. The SLOE in EM is a step toward decreasing bias through structured reporting of performance assessment; however, it is not entirely free from it.^82^,^83^ Narrative evaluations for men are more supportive than for women and UIM students.^84^,^85^ The UIM students with similar clerkship grades had more negative comments and fewer positive comments compared to their White counterparts.^86^ The SLOE is often perceived as the most objective assessment of the student’s clinical competency and potential as a rising resident; however, the influence of bias in EM SLOE rankings and language has been insufficiently studied.^87^,^88^

Traditional letters of recommendation are often reflective of a student’s network and support system, which may be more difficult to develop for women and UIM students. They are also subject to language bias. Women and UIM students are more likely to be characteried by grindstone words such as “organized” or “hardworking” as compared to superlatives reflecting high achievement potential used in letters for White male students.^9^,^62^,^84^,^87^,^89^–^91^

#### Medical Student Performance Evaluation

The MSPE is a comprehensive review of a student’s interests, activities, and, most notably, clinical performance. Some schools provide rankings of the student in comparison to their peers. Only 2% of medical schools provide comparative data consistently in all five appendices (pre-clinical courses, clerkships, professional attributes, overall performance, and medical school information page).^88^,^92^ Furthermore, the MSPE can be fraught with the use of biased language and descriptions based on the applicant’s gender^84^ and race/ethnicity.^85^,^90^,^93^ White students were more likely to be described as “outstanding,” “exceptional,” and “best.”^93^ In contrast, the word “competent” was more often used to describe Black and Hispanic students but was only perceived to carry a positive connotation 37% and 33% of the time, respectively.^93^

#### Alpha Omega Alpha

Acceptance into AOA is often used to signal academic excellence. However, awards and accolades have been shown to be given less often to UIM students, and not all institutions participate in AOA.^88^ Membership in AOA was six times more likely for White students than for Black students.^94^ Use of this award as a differentiating factor can be discriminatory and disadvantage UIM students.^95^

#### Standardized Exams

Standardized examinations have been shown to predict academic success on in-training and board exams but not to predict success in residency or an ability to provide safe and quality care overall.^46^,^74^,^96^,^97^ Despite this, USMLE Step 1 scores are commonly used as a screening tool.^9^,^11^,^62^,^97^ The USMLE is subject to systemic biases associated with any standardized test, such as accessibility and affordability of test prep. Given that UIM applicants have lower USMLE scores on average,^98^,^99^ an over-reliance on test scores as a screening tool can lead to UIM applicants being excluded from a more in-depth review that may have otherwise earned them an interview invitation.^11^,^12^,^26^,^52^,^62^,^72^,^98^,^100^ In 2020, it was announced that the USMLE Step 1 exam will be scored as pass or fail based on previous evidence of poor utility. The USMLE Step 2, as well as other standardized exams such as the Comprehensive Osteopathic Medical Licensing Examination, will still report numerical scores.^101^

#### Personal Statement

Personal statements allow applicants to share stories of inspiration, resilience, and future goals,^102^ enabling them to showcase their interests and skills. The value placed on the personal statement is variable, however,^103^ and may introduce bias such as gender-based differences in writing.^104^ Personal statements are not effective in predicting medical student performance,^105^ and utility for residency selection is unclear.

### Interview Process and Considerations

The interview allows for scoring on behavioral-related metrics, such as grit, distance traveled in life experience, and emotional intelligence.^106^ Steps should be taken to standardize the interview process as much as possible to minimize bias.^46^,^107^ The interview should follow a set structure. A standard pool of questions should be determined ahead of time and interviewers can be assigned specific questions.^78^ Interviewers should receive the same instructional training and have access to the same amount of information from applications.^30^,^44^,^54^

As performance metrics can bias perceptions,^108^ programs should consider blinded interviews in which exam scores are not provided to interviewers.^109^ Interviewers should represent a diverse pool of faculty, residents, and staff and should receive protected time to support the commitments needed for thoughtful interviewing.^30^ The COVID-19 pandemic required that the 2020 recruitment cycle be done virtually. Programs should decide whether they will offer virtual or in-person interviews, and all interviews should be done in the same format to avoid bias.^110^

#### Travel considerations

The UIM trainees experience greater financial challenges from the high cost of medical education.^111^,^112^ Digital interviewing contributed to less financial burden from traveling. The emphasis on away rotations in EM, however, creates a potential hurdle for UIM applicants. Clerkship diversity scholarships have been shown to correlate with increased residency diversity in EM, especially for Black and Latino residents.^113^ Scholarships and financial assistance can attract UIM applicants who otherwise would be unable to rotate at, and may not have considered, a particular program. Funding for UIM recruitment efforts demonstrate institutional commitment to diversity recruitment.^50^,^51^

#### Consideration for Historically Black Colleges and Universities

Students from historically Black colleges and universities (HBCU) often rely on away rotations for their sub-internship experience as their home institution may have limited exposure to EM or lack an emergency department. The ability to fund travel and lodging limits the options of rotation location.^112^ If students are unable to travel due to financial restrictions, their opportunity to be exposed to new clinical environments and potential mentors is limited.^50^,^51^,^113^ Partnerships between HBCUs and neighboring EM residency programs can help promote diversity.^114^

**Best Practice Recommendations**

1. Apply an equity lens to each step of the recruitment process to expose existing bias and allow for correction. (Level 5, Grade D)
2. Holistic review should be applied equitably across all applicants. (Level 4, Grade C)
3. Identify characteristics for holistic review that align with a program’s mission, vision, values. (Level 4, Grade C)
4. Avoid screening applicants solely on standardized examination scores or grades. (Level 3b, Grade C)
5. Standardize the structure of interviews in terms of logistics and questions asked. (Level 4. Grade C)
6. Ensure UIM faculty visibility and allow networking during the interview day or through structured asynchronous opportunities to engage with DEI topics. (Level 4, Grade C)
7. Partner with HBCUs and neighboring EM residency programs to help further promote diversity within the specialty. (Level 4, Grade C)

## POST-INTERVIEW STRATEGIES

### Ranking Considerations

The ranking process should be collaborative and conducted in a safe space with limited external influence from those not involved in the recruitment process.^115^ Members of the ranking group should be diverse in interests and backgrounds and often include the residency leadership team as well as additional core faculty, residents, and administrative staff. Individuals participating in rank meetings should be informed about characteristics identified of value to the residency program and used throughout the selection process.^11^,^25^,^50^,^51^ One voice should not dominate, and there should be group discussion prior to deciding a rank position. A temporary ranking meeting might occur immediately following the day of an interview. For fairness to all candidates, the final ranking should begin at the conclusion of the interview season.^12^,^116^

### Second-look Opportunities and Post-interview Communication

There should be increased visibility and opportunity to network with faculty (both UIM and non-UIM) involved in recruitment and retention of UIM applicants.^25^,^50^ If UIM recruitment faculty are not available during scheduled interview dates, asynchronous opportunities to discuss DEI within the program should be offered.^51^ A second-look visit can be organized to facilitate this.^23^,^50^

Targeted recruitment of UIM applicants may benefit from ongoing dialogue throughout the interview process. Communication such as “thank you” emails should be done with heavy consideration of the potential to mislead or falsely assure an applicant. Caution should be taken when reaching out to UIM applicants to not breach National Resident Matching Program regulations. Programs should be clear about expectations for post-interview communication and should designate a pointperson for ongoing communication.

**Best Practice Recommendations**

1. Select diverse members for the rank committee. (Level 4, Grade C)
2. Conduct the rank meeting in a safe, private space with collaborative discussion. (Level 4, Grade C)
3. Inform committee members about the characteristics identified as valuable to the program before the ranking process. (Level 5, Grade D)
4. Ensure ranking is done based on scores from the predefined rubrics for screening and interviewing. (Level 5, Grade D)
5. Offer second look visits (on-site or virtually) to network with UIM faculty and discuss DEI within the program. (Level 4, Grade C)
6. Define clear expectations for follow up and designate a point person for communication. (Level 4, Grade C)

## LIMITATIONS

The scope of this article was limited to holistic review and the impact of bias on recruitment in residency training. There are other topics (eg, pipeline/pathway efforts, faculty recruitment and retention) regarding DEI that will be covered in other reviews. While we performed a comprehensive search guided by a medical librarian in conjunction with expert consultation and bibliographic review, it is possible that we may have missed pertinent articles. In several instances, high-quality data was limited or lacking. In these instances, we relied upon expert opinion and group consensus for the best practice recommendations. Literature specific to EM and within graduate medical education is more limited; therefore, we included relevant articles from other medical specialties and health-related professions. We believe that EM, as a specialty, can learn from other colleagues across many disciplines.

## CONCLUSION

Holistic review and the mitigation of bias are essential steps in the purposeful recruitment and selection of applicants who are underrepresented in medicine. Our article presents best practice recommendations for residency programs to prepare for and implement application review, applicant interviewing, and trainee selection in support of diversity, equity, and inclusion.

## Supplementary Information





## Figures and Tables

**Table 1a:** Oxford Centre for Evidence-Based Medicine levels of evidence.^20^

Level of evidence	Definition
1a	Systematic review of homogenous RCTs
1b	Individual RCT
2a	Systematic review of homogenous cohort studies
2b	Individual cohort study or a low-quality RCT*
3a	Systematic review of homogenous case-control studies
3b	Individual case-control study**
4	Case series/Qualitative studies or low-quality cohort or case-control study***
5	Expert/consensus opinion

* defined as <80% follow up;

** includes survey studies and cross-sectional studies;

*** defined as studies without clearly defined study groups.

*RCT*, randomized controlled trial.

**Table 1b:** Oxford Centre for Evidence-Based Medicine grades of recommendation.^20^

Grade of evidence	Definition
A	Consistent level 1 studies
B	Consistent level 2 or 3 studies or extrapolations* from level 1 studies
C	Level 4 studies or extrapolations* from level 2 or 3 studies
D	Level 5 evidence or troublingly inconsistent or inconclusive studies of any level

* “Extrapolations” refer to the use of data in a situation that has potentially clinically important differences from the original study situation.

**Table 2:** Key steps to assessing culture and climate comprehensively.*

Step	Application
Reflection	Reflective questions for personal exploration on relevant criteria
Data Collection	Data collection processes and tools to capture the determinants of the culture of diversity and inclusion
Synthesis and Analysis	Synthesis and analysis to identify areas of strength and opportunities
Leverage Findings	Leverage findings to translate assessment findings into institutional outcomes

* Adapted from the Association of American Medical Colleges.^35^

**Table 3:** Example scoring rubrics incorporating holistic review concepts.

Reference	Specialty	Considerations
UCSF GME Handbook for Holistic Review and Best Practices for Enhancing Diversity in Residency and Fellowship Programs^81^	Internal Medicine	Uses a Likert scale of 1–5 to provide scores for components from file review, interview observations, and as an overall rating. File review carries more weight than the interview.
DeBenedectis 2019^12^	Radiology	USMLE Step 1 and medical school grades/ranking are only 2 of 10 items scored and are given the same value as other factors (0–3 points each). Factors known to be less associated with diversity, such as research and publications, continue to be included.
Aibana 2019^78^	Internal Medicine	Experience/attribute score is calculated if the applicant does not meet USMLE cutoff score but is within 10 points, creating an opportunity to “rescue” an applicant and still offer an interview. USMLE scores are still used for screening.
Barcelo 2021^79^	Psychiatry	Use of a positive multiplier if resilience or distance traveled was noticed. Domains and clusters of characteristics with varying tiers of significance create complex composite scores.

*UCSF*, University of California - San Francisco; *GME*, graduate medical education; *USMLE*, United States Medical Licensing Exam.

## Footnotes

a Defined by the Association of American Medical Colleges (AAMC), under-represented minority (URM) was a term reflecting the racial groups of Black, Mexican-American, mainland Puerto Rican, and Native American. (American Indian and natives of Alaska and Hawaii). This was expanded to “underrepresented in medicine”(URiM or UIM) in 2003 to encompass the racial and ethnic populations within medicine who are underrepresented when compared to their respective numbers in the context of the greater population. For this article, although we recognize the use of URiM or UIM interchangeably, for consistency, we use the term UIM.
