# Supplementary material for: Holistic Review, Mitigating Bias, and Other Strategies in Residency Recruitment for Diversity, Equity, and Inclusion: An Evidence-based Guide to Best Practices from the Council of Residency Directors in Emergency Medicine
Source: West J Emerg Med. 2022 May 10;23(3):345–52. doi: 10.5811/westjem.2022.3.54419 (PMC9183777; doi:10.5811/westjem.2022.3.54419)
Supplement: Supplementary file 1 [file wjem-23-345-s001.docx]

# **APPENDIX. Search Strategy**

**PubMed (Date of Search: 1/21/21)**

(((medical education OR meded[tiab]) AND (recruitment OR recruit* OR retention[tiab] OR retain* OR pipeline)) AND (diversity OR diverse OR inclusive OR underrepresented OR minority OR minorities OR ethnic OR ethnicity OR ethnicities OR racial OR race OR tokenism OR token[tiab] OR Black OR Asian OR Blacks OR Asians OR Puerto Rican OR Mexican American OR Native American OR American Indian OR Alaskan Native OR Hawaiian OR African American OR Hispanic OR Latino OR Latinx OR Latina)) AND (physician OR doctor OR trainee OR residency OR trainees OR residency OR interns OR intern OR faculty)
