# Supplementary material for: Holistic Review, Mitigating Bias, and Other Strategies in Residency Recruitment for Diversity, Equity, and Inclusion: An Evidence-based Guide to Best Practices from the Council of Residency Directors in Emergency Medicine
Source: West J Emerg Med. 2022 May 10;23(3):345–52. doi: 10.5811/westjem.2022.3.54419 (PMC9183777; doi:10.5811/westjem.2022.3.54419)
Supplement: Supplementary file 2 [file wjem-23-345-s002.docx]

**REFERENCES**

1. Arno K, Davenport D, Shah M, et al. Addressing the urgent need for racial diversification in emergency medicine. *Ann Emerg Med*. 2021;77(1):69-75.
2. Heron SL, Lovell EO, Wang E, et al. Promoting diversity in emergency medicine: summary recommendations from the 2008 Council of Emergency Medicine Residency Directors (CORD) Academic Assembly Diversity Workgroup. *Acad Emerg Med.* 2009;16(5):450–3.
3. Whitla DK, Orfield G, Silen W, et al. Educational benefits of diversity in medical school: a survey of students. *Acad Med.* 2003;78(5):460–6.
4. Abelson JS, Symer MM, Yeo HL, et al. Surgical time out: Our counts are still short on racial diversity in academic surgery. *Am J Surg.* 2018;215(4):542–8.
5. Bennett CL, Yiadom MYAB, Baker O, et al. Examining parity among Black and Hispanic resident physicians. *J Gen Intern Med.* 2021;36(6):1722-1725.
6. Gonzaga AMR, Appiah-Pippim J, Onumah CM, et al. A framework for inclusive graduate medical education recruitment strategies: meeting the ACGME Standard for a Diverse and Inclusive Workforce. *Acad Med*. 2020;95(5):710–6.
7. Thomas BR, Dockter N. Affirmative Action and holistic review in medical school admissions: Where we have been and where we are going. *Acad Med.* 2019;94(4):473–6.
8. Person SD, Jordan CG, Allison JJ, et al. Measuring diversity and inclusion in academic medicine: the Diversity Engagement Survey. *Acad Med*. 2015;90(12):1675–83.
9. Angus SV, Williams CM, Stewart EA, et al. Internal medicine residency program directors’ screening practices and perceptions about recruitment challenges. *Acad Med.* 2020;95(4):582–9.
10. Boatright D, Branzetti J, Duong D, et al. Racial and ethnic diversity in academic emergency medicine: How far have we come? Next steps for the future. *AEM Educ Train*. 2018;2(Suppl Suppl 1):S31–9.
11. Clayborne EP, Martin DR, Goett RR, et al. Diversity pipelines: the rationale to recruit and support minority physicians. *J Am Coll Emerg Physicians Open*. 2021;2(1)e12343.
12. DeBenedectis CM, Heitkamp DE, England E, et al. A program director’s guide to cultivating diversity and inclusion in radiology residency recruitment. *Acad Radiol*. 2020;27(6):864–7.
13. Nakae S, Porfeli EJ, Davis D, et al. Enrollment management in undergraduate medical school admissions: a complementary framework to holistic review for increasing diversity in medicine. *Acad Med*. 2021;96(4):501-6.
14. Gottlieb M, King A, Byyny R, et al. Journal club in residency education: an evidence-based guide to best practices from the Council of Emergency Medicine Residency Directors. *West J Emerg Med.* 2018;19(4):746–55.
15. Estes M, Gopal P, Siegelman JN, et al. Individualized interactive instruction: a guide to best practices from the Council of Emergency Medicine Residency Directors. *West J Emerg Med*. 2019;20(2):363–8.
16. Parsons M, Bailitz J, Chung AS, et al. Evidence-based interventions that promote resident wellness from the Council of Emergency Residency Directors. *West J Emerg Med*. 2020;21(2):412–22.
17. Natesan S, Bailitz J, King A, et al. Clinical teaching: an evidence-based guide to best practices from the Council of Emergency Medicine Residency Directors. *West J Emerg* *Med*. 2020;21(4):985–98.
18. Wood DB, Jordan J, Cooney R, et al. Conference didactic planning and structure: an evidence-based guide to best practices from the Council of Emergency Medicine Residency Directors. *West J Emerg Med*. 2020;21(4):999-1007.
19. Chathampally Y, Cooper B, Wood DB, et al. Evolving from morbidity and mortality to a case-based error reduction conference: evidence-based best practices from the Council of Emergency Medicine Residency Directors. *West J Emerg Med.* 2020;21(6):231–41.
20. Davenport D, Alvarez A, Natesan S, et al. Faculty recruitment, retention, and representation in leadership: an evidence-based guide to best practices for diversity, equity, and inclusion from the Council of Residency Directors in Emergency Medicine. *West J Emerg Med*. 2022;23(1):62-71.
21. Howick J, Chalmers I, GlasziouP, et al. Explanation of the 2011 Oxford Centre for Evidence-Based Medicine (OCEBM) levels of evidence (Background Document). Available at: <https://www.cebm.ox.ac.uk/resources/levels-of-evidence/ocebm-levels-of-evidence>. Accessed August 13, 2021.
22. Raphael JL, Giardino AP, Harris T, et al. Perceptions revisited: pediatric chief resident views on minority housestaff recruitment and retention in pediatric residency programs. *J Natl Med Assoc.* 2014;106(1):58–68.
23. Pierre JM, Mahr F, Carter A, et al. Underrepresented in medicine recruitment: rationale, challenges, and strategies for increasing diversity in psychiatry residency programs. *Acad Psychiatry.* 2017;41(2):226–32.
24. Vick AD, Baugh A, Lambert J, et al. Levers of change: a review of contemporary interventions to enhance diversity in medical schools in the USA. *Adv Med Educ Pract*. 2018;9:53–61.
25. Lewis T, Tolbert J, Jones BL. Increasing resident racial and ethnic diversity through targeted recruitment efforts. *J Pediatr*. 2020;216:4–6.
26. Jarman BT, Kallies KJ, Joshi ART, et al. Underrepresented minorities are underrepresented among general surgery applicants selected to interview. *J Surg Educ*. 2019;76(6):e15–23.
27. Lim RF, Luo JS, Suo S, et al. Diversity initiatives in academic psychiatry: applying cultural competence. *Acad Psychiatry*. 2008;32(4):283–90.
28. Ko M, Ton H. The not underrepresented minorities: Asian Americans, diversity, and admissions. *Acad Med*. 2020;95(2):184–9.
29. Toney M. The long, winding road: one university’s quest for minority health care professionals and services. *Acad Med.* 2012;87(11):1556–61.
30. Mateo CM, Williams DR. More than words: a vision to address bias and reduce discrimination in the health professions learning environment. *Acad Med*. 2020;95(12S Addressing harmful bias and eliminating discrimination in health professions learning environments):S169–77.
31. Deas D, Pisano E, Mainous A, et al. Improving diversity through strategic planning: a 10-year (2002-2012) experience at the Medical University of South Carolina. *Acad Med*. 2012;87(11):1548–55.
32. Heron S, Haley L Jr. Diversity in emergency medicine--a model program. *Acad Emerg Med*. 2001;8(2):192–5.
33. Mmeje O, Price EAN, Johnson TRB, et al. Galvanizing for the future: a bottom-up departmental approach to diversity, equity, and inclusion. *Am J Obstet Gynecol*. 2020;223(5):715.e1-715.e7.
34. Rymer JA, Frazier-Mills CG, Jackson LR, et al. Evaluation of women and underrepresented racial and ethnic group representation in a general cardiology fellowship after a systematic recruitment initiative. *JAMA Netw Open.* 2021;4(1):e2030832.
35. Assessing Institutional Culture and Climate Webcast Supplemental Guide 2013. Assoc Am Med Coll 2013;27.
36. Sanson-Fisher RW, Williams N, Outram S. Health inequities: the need for action by schools of medicine. *Med Teach.* 2008;30(4):389–94.
37. Adetoye M. Recruitment and retention: a guideline to help build trust with minority candidates. *Acad Med.* 2019;94(6):754.
38. Boatright D, Tunson J, Caruso E, et al. The impact of the 2008 Council of Emergency Residency Directors (CORD) Panel on Emergency Medicine Resident Diversity. *J Emerg Med*. 2016;51(5):576–83.
39. Jarman BT, Borgert AJ, Kallies KJ, et al. Underrepresented minorities in general surgery residency: analysis of interviewed applicants, residents, and core teaching faculty. *J Am Coll Surg*. 2020;231(1):54–8.
40. Okike K, Phillips DP, Johnson WA, et al. Orthopaedic faculty and resident racial/ethnic diversity is associated with the orthopaedic application rate among underrepresented minority medical students. *J Am Acad Orthop Surg*. 2020;28(6):241–7.
41. Johnson TJ, Ellison AM, Dalembert G, et al. Implicit bias in pediatric academic medicine. *J Natl Med Assoc.* 2017;109(3):156–63.
42. Capers IV Q. How clinicians and educators can mitigate implicit bias in patient care and candidate selection in medical education. *ATS* *Sch*. 2020;1(3):211–7.
43. Shappell E, Schnapp B. The F word: how “fit” threatens the validity of resident recruitment. *J Grad Med Educ.* 2019;11(6):
    635–6.
44. Spottswood SE, Spalluto LB, Washington ER, et al. Design, implementation, and evaluation of a diversity program for radiology. *J Am Coll Radiol*. 2019;16(7):983–91.
45. Cleveland Manchanda EC, Macias-Konstantopoulos WL. Tackling gender and racial bias in academic emergency medicine: the perceived role of implicit bias in faculty development. *Cureus.* 2020;12(11):e11325.
46. Spector AR, Railey KM. Reducing reliance on test scores reduces racial bias in neurology residency recruitment. *J Natl Med Assoc.* 2019;111(5):471–4.
47. Girod S, Fassiotto M, Grewal D, et al. Reducing implicit gender leadership bias in academic medicine with an educational intervention. *Acad Med*. 2016;91(8):1143–50.
48. Paterson QS, Hartmann R, Woods R, et al. A transparent and defensible process for applicant selection within a Canadian emergency medicine residency program. *Can J Emerg Med*. 2020;22(2):215–23.
49. Kiraly L, Dewey E, Brasel K. Hawks and doves: adjusting for bias in residency interview scoring. *J Surg Educ*. 2020;77(6):e132–7.
50. Tunson J, Boatright D, Oberfoell S, et al. Increasing resident diversity in an emergency medicine residency program: a pilot intervention with three principal strategies. *Acad Med*. 2016;91(7):958–61.
51. Winfield A, Schindlbeck M, Smith L. Additional strategies for underrepresented minority recruitment. *Ann Emerg Med.* 2020;75(2):313–4.
52. Garrick JF, Perez B, Anaebere TC, et al. The diversity snowball effect: the quest to increase diversity in emergency medicine: a case study of Highland’s emergency medicine residency program. *Ann Emerg Med.* 2019;73(6):639–47.
53. Vajapey S, Cannada LK, Samora JB. What proportion of women who received funding to attend a Ruth Jackson Orthopaedic Society meeting pursued a career in orthopaedics? *Clin Orthop Relat Res*. 2019;477(7):1722–6.
54. Wusu MH, Tepperberg S, Weinberg JM, et al. Matching our mission: a strategic plan to create a diverse family medicine residency. *Fam Med.* 2019;51(1):31–6.
55. Van Voorhees AS, Enos CW. Diversity in dermatology residency programs. *J Investig Dermatol* *Symp Proc*. 2017;18(2):S46-s49.
56. Lin MP, Lall MD, Samuels-Kalow M, et al. Impact of a women-focused professional organization on academic retention and advancement: perceptions from a qualitative study. *Acad Emerg Med*. 2019;26(3):303–16.
57. Nivet MA, Berlin A. Workforce diversity and community-responsive health-care institutions. *Public Health Rep*. 2014;129 Suppl 2(Suppl 2):15–8.
58. Muppala VR, Prakash N. Promoting physician diversity through medical student led outreach and pipeline programs. *J Natl Med Assoc*. 2021;113(2):165-168.
59. Kumar V, West DL. Bridging the equity gap. *AJR Am J Roentgenol*. 2019;213(4):785–91.
60. Lievens F. Diversity in medical school admission: insights from personnel recruitment and selection. *Med Educ.* 2015;49(1):11–4.
61. Rumala BB, Cason FD Jr. Recruitment of underrepresented minority students to medical school: minority medical student organizations, an untapped resource. *J Natl Med Assoc.* 2007;99(9):1000–4, 1008.
62. Gerull KM, Enata N, Welbeck AN, et al. Striving for inclusive excellence in the recruitment of diverse surgical residents during COVID-19. *Acad Med*. 2021;96(2):210–2.
63. Common Program Requirements (Residency). *ACGME.* 2020. Available at: <https://www.acgme.org/Portals/0/PFAssets/ProgramRequirements/CPRResidency2021.pdf>. Accessed August 13, 2021.
64. Poole KG Jr, Jordan BL, Bostwick JM. Mission drift: Are medical school admissions committees missing the mark on diversity? *Acad Med.* 2020;95(3):357–60.
65. Nehemiah A, Roberts SE, Song Y, et al. Looking beyond the numbers: increasing diversity and inclusion through holistic review in general surgery recruitment. *J Surg Educ*. 2021;78(3):763–9.
66. Williams C, Kwan B, Pereira A, Moody E, et al. A call to improve conditions for conducting holistic review in graduate medical education recruitment. *MedEdPublish*. 2019;8(2):6.
67. Crane JT, Ferraro CM. Selection criteria for emergency medicine residency applicants. *Acad Emerg Med*. 2000;7(1):54–60.
68. Garber AM, Kwan B, Williams CM, et al. Use of filters for residency application review: results from the Internal Medicine In-Training Examination Program Director Survey. *J Grad Med Educ* 2019;11(6):704–7.
69. Association of American Medical Colleges. Holistic review. Available at: <https://www.aamc.org/services/member-capacity-building/holistic-review>. Accessed August 13, 2021.
70. Association of American Medical Colleges. Applicant Criteria Identification and Prioritization. Available at: <https://www.aamc.org/media/44576/download>. Accessed August 13, 2021.
71. Patterson F, Roberts C, Hanson MD, et al. 2018 Ottawa consensus statement: Selection and recruitment to the healthcare professions. *Med Teach.* 2018;40(11):1091–101.
72. Damp JB, Cullen MW, Soukoulis V, et al. Program directors survey on diversity in cardiovascular training programs. *J Am Coll Cardiol*. 2020;76(10):1215–22.
73. Witzburg RA, Sondheimer HM. Holistic review--shaping the medical profession one applicant at a time. *N Engl J Med*. 2013;368(17):1565–7.
74. Shantharam G, Tran TY, McGee H, et al. Examining trends in underrepresented minorities in urology residency. *Urology*. 2019;127:36–41.
75. Fassiotto M, Hamel EO, Ku M, et al. Women in academic medicine: measuring stereotype threat among junior faculty. *J Womens Health*. 2016;25(3):292–8.
76. Dossett LA, Mulholland MW, Newman EA, et al. Building high-performing teams in academic surgery: the opportunities and challenges of inclusive recruitment strategies. *Acad Med.* 2019;94(8):1142–5.
77. Marbin J, Rosenbluth G, Brim R, et al. Improving diversity in pediatric residency selection: using an equity framework to implement holistic review. *J Grad Med Educ*. 2021;13(2):195–200.
78. Aibana O, Swails JL, Flores RJ, et al. Bridging the gap: holistic review to increase diversity in graduate medical education. *Acad Med*. 2019;94(8):1137–41.
79. Barceló NE, Shadravan S, Wells CR, et al. Reimagining merit and representation: promoting equity and reducing bias in GME through holistic review. *Acad Psychiatry*. 2021;45(1):34–42.
80. American Association of Medical Colleges. Holistic Principles in Resident Selection: An Introduction. 2020*.* Available at: <https://www.aamc.org/system/files/2020-08/aa-member-capacity-building-holistic-review-transcript-activities-GME-081420.pdf>. Accessed August 13, 2021.
81. UCSF Graduate Medical Education. Handbook for Holistic Review and Best Practices for Enhancing Diversity in Residency and Fellowship Programs. 2017. Available at: <https://wiki.library.ucsf.edu/download/attachments/456075181/Holistic%20Review%20Best%20Practices%209.21.17-FINAL.pdf?version=1&modificationDate=1592508759000&api=v2>. Accessed August 13, 2021.
82. Love JN, Doty CI, Smith JL, et al. The Eeergency medicine group Standardized Letter of Evaluation as a workplace-based assessment: the validity is in the detail. *West J Emerg Med*. 2020;21(3):600–9.
83. Wilson D, Laoteppitaks C, Chandra S. A comparison of Standardized Letters of Evaluation for emergency medicine residency applicants. *West J Emerg Med*. 2020;22(1):20–5.
84. Hewett L, Lewis M, Collins H, et al. Gender bias in diagnostic radiology resident selection, Does it exist? *Acad Radiol*. 2016;23(1):101–7.
85. Low D, Pollack SW, Liao ZC, et al. Racial/ethnic disparities in clinical grading in medical school. *Teach Learn Med.* 2019;31(5):487–96.
86. Lee KB, Vaishnavi SN, Lau SKM, et al. Cultural competency in medical education: demographic differences associated with medical student communication styles and clinical clerkship feedback. *J Natl Med Assoc.* 2009;101(2):116–26.
87. Miller DT, McCarthy DM, Fant AL, et al. The Standardized Letter of Evaluation narrative: differences in language use by gender. *West J Emerg Med.* 2019;20(6):948–56.
88. Pope AJ, Carter K, Ahn J. A renewed call for a more equitable and holistic review of residency applications in the era of COVID-19. *AEM Educ Train.* 2021;5(1):135–8.
89. Heath JK, Weissman GE, Clancy CB, et al. Assessment of gender-based linguistic differences in physician trainee evaluations of medical faculty using automated text mining. *JAMA Netw Open*. 2019;2(5):e193520.
90. Diaz T, Navarro JR, Chen EH. An institutional approach to fostering inclusion and addressing racial bias: implications for diversity in academic medicine. *Teach Learn Med*. 2020;32(1):110–6.
91. Filippou P, Mahajan S, Deal A, et al. The presence of gender bias in letters of recommendations written for urology residency applicants. *Urology*. 2019;134:56–61.
92. Boysen-Osborn M, Yanuck J, Mattson J, et al. Who to interview? Low adherence by U.S. medical schools to medical student performance evaluation format makes resident selection difficult. *West J Emerg Med.* 2017;18(1):50–5.
93. Ross DA, Boatright D, Nunez-Smith M, et al. Differences in words used to describe racial and gender groups in Medical Student Performance Evaluations. *PLOS ONE*. 2017;12(8):e0181659.
94. Boatright D, Ross D, O’Connor P, et al. Racial disparities in medical student membership in the Alpha Omega Alpha Honor Society. *JAMA Intern Med*. 2017;177(5):659.
95. Boatright D, O’Connor PG, E Miller J. Racial privilege and medical student awards: addressing racial disparities in Alpha Omega Alpha Honor Society membership. *J Gen Intern Med*. 2020;35(11):3348–51.
96. Lypson ML, Ross PT, Hamstra SJ, et al. Evidence for increasing diversity in graduate medical education: the competence of underrepresented minority residents measured by an intern objective structured clinical examination. *J Grad Med Educ*. 2010;2(3):354–9.
97. Prober CG, Kolars JC, First LR, et al. A plea to reassess the role of United States Medical Licensing Examination Step 1 scores in residency selection. *Acad Med*. 2016;91(1):12–5.
98. Williams M, Kim EJ, Pappas K, et al. The impact of United States Medical Licensing Exam (USMLE) Step 1 cutoff scores on recruitment of underrepresented minorities in medicine: a retrospective cross-sectional study. *Health Sci Rep*. 2020;3(2):e2161.
99. Youmans QR, Essien UR, Capers Q. A test of diversity - what USMLE pass/fail scoring means for medicine. *N Engl J Med.* 2020;382(25):2393–5.
100. Lucey CR, Saguil A. The consequences of structural racism on MCAT scores and medical school admissions: The past Is prologue. *Acad Med.* 2020;95(3):351–6.
101. Makhoul AT, Pontell ME, Kumar NG, et al. Objective measures needed — program directors’ perspectives on a pass/fail USMLE Step 1. *N Engl J Med*. 2020;382(25):2389-92.
102. Melamed KH, Wang T. The personal statement: not just a question of if it is valued, but should it be valued? *ATS Sch*. 2020;1(1):5–7.
103. Hinkle L, Carlos WG, Burkart KM, et al. What do program directors value in personal statements? A qualitative snalysis. *ATS Sch*. 2020;1(1):44–54.
104. Ostapenko L, Schonhardt-Bailey C, Sublette JW, et al. Textual analysis of general surgery residency personal statements: topics and gender differences. *J Surg Educ*. 2018;75(3):573–81.
105. Patterson F, Knight A, Dowell J, et al. How effective are selection methods in medical education? A systematic review. *Med Educ.* 2016;50(1):36–60.
106. Pritchett EN, Pandya AG, Ferguson NN, et al. Diversity in dermatology: roadmap for improvement. *J Am Acad Dermatol*. 2018;79(2):337–41.
107. Hughes RH, Kleinschmidt S, Sheng AY. Using structured interviews to reduce bias in emergency medicine residency recruitment: worth a second look. *AEM Educ Train*. 2021;5(Suppl 1):S130-S134.
108. Stephenson-Famy A, Houmard BS, Oberoi S, et al. Use of the interview in resident candidate selection: a review of the literature. *J Grad Med Educ.* 2015;7(4):539–48.
109. Fuchs JW, Youmans QR. Mitigating bias in the era of virtual residency and fellowship interviews. *J Grad Med Educ.* 2020;12(6):674–7.
110. Deitte LA, Mian AZ, Esfahani SA, et al. Going virtual: redesigning the interview experience. *J Am Coll Radiol.* 2020;18(2):337–9.
111. Davis MG, Haas MRC, Gottlieb M, et al. Zooming In versus flying out: virtual residency interviews in the era of COVID-19. *AEM Educ Train.* 2020;4(4):443–6.
112. Toretsky C, Mutha S, Coffman J. Breaking barriers for underrepresented minorities in the health professions. 2018. Available at: <https://healthforce.ucsf.edu/publications/breaking-barriers-underrepresented-minorities-health-professions>. Accessed August 13, 2021.
113. Duong DK, Samuels EA, Boatright D, et al. Association between emergency medicine clerkship diversity scholarships and residency diversity. *AEM Educ Train*. 202;5(3):e10547.
114. Goines J, Iledare E, Ander D, et al. A model partnership: mentoring underrepresented students in medicine (URiM) in emergency medicine. *West J Emerg* *Med*. 2021;22(2):213–7.
115. Young ME, Thomas A, Varpio L, et al. Facilitating admissions of diverse students: a six-point, evidence-informed framework for pipeline and program development. *Perspect Med Educ*. 2017;6(2):82–90.
116. Fryer JP, Corcoran N, George B, Wang E, Darosa D. Does resident ranking during recruitment accurately predict subsequent performance as a surgical resident? *J Surg Educ*. 2012;69(6):724–30
